# Evidence-based approaches for advancing clinical research and practice: dentistry beyond the myth

**DOI:** 10.1590/1807-3107bor-2026.vol40suppl1057

**Published:** 2026-08-24

**Authors:** Lucianne Cople MAIA

**Affiliations:** (a) Universidade Federal do Rio de Janeiro – UFRJ, School of Dentistry, Department of Pediatric Dentistry and Orhodontics, Rio de Janeiro, RJ, Brazil

Throughout history, the generation of knowledge has assumed different roles, functions, and perspectives. We have evolved from a period in which knowledge was largely based on unsystematic observation, personal experience, and limited methodological control to the consolidation of the scientific method. Through this evolution, the collection, analysis, and interpretation ofdata have become the foundation for producing the best available evidence across different fields ofknowledge.

However, in today’s globalized scientific context, an overwhelming amount of information circulates rapidly, often without adequate organization or critical appraisal. In this environment, unsupported or distorted claims may be repeatedly disseminated until they become widely accepted as scientific facts, leading to persistence of myths that are difficult to challenge.

Guided by the view of science as a dynamic and rigorous process, this Special Issue brings together leading national and international experts to examine relevant topics in Dentistry. It also aims to clarify misconceptions and challenge persistent myths, by presenting current evidence on risk factors, diagnosis, treatment, and prognosis across several dental specialties to support better research practices and clinical decisions.

One of these contributions reviews current evidence and future directions for artificial intelligence-assisted diagnostic workflows in selected oral conditions, including odontogenic cysts, odontogenic tumors, oral potentially malignant disorders, and oral squamous cell carcinoma.^1^ Another article examines laser applications in Dentistry, focusing on photobiomodulation mechanisms, dosimetry and tissue interactions. It also addresses the clinical management of oral pain disorders, oncology-related complications, immune-mediated diseases, and viral infections.^2^

Misconceptions about restorative “bioactivity” in clinical practice are also addressed. By discussing how bioactive materials interact with the dentin-pulp complex and influences mineral dynamics, matrix stability, and cellular responses, the article emphasizes that the “bioactive” label requires evidence that these materials can sustain dentin-pulp homeostasis rather than merely replace lost tooth structure.^3^

A further contribution discusses the management of molar-incisor hypomineralization (MIH), including its diagnosis and severity assessment. The prevention of enamel breakdown, restoration ofseverely affected molars, esthetic approaches to mask anterior enamel opacities, treatment of hypersensitivity were throughout discussed.^4^

As with the other topics addressed, dental bleaching remains surrounded by misconceptions and is examined in one article. Using examples from cognitive and social psychology to explain why myths may promote inappropriate, scientifically unsupported practices, the authors revisit key bleaching concepts and clinical practices, challenging common myths and summarizing criteria for selecting appropriate bleaching protocols.^5^

Regenerative endodontics has transformed the management of immature necrotic teeth by offering the potential to promote continued root development rather than merely inducing apical closure. A comprehensive synthesis of the current evidence discusses contemporary clinical protocols, prognostic factors, treatment outcomes, existing limitations, and emerging therapeutic strategies, providing an updated perspective on the current status and future directions of regenerative endodontics.^6^

Periodontal diseases and peri-implantitis are addressed in two other articles.^7^, ^8^ Both diseases are recognized as multifactorial conditions influenced by biological, behavioral, and environmental factors. The role of cigarette smoking is a well-established risk factor for periodontitis raising questions about whether electronic cigarettes, which are increasingly used as alternatives to combustible tobacco, may also affect periodontal health. Thus, one article^7^ critically appraises the available evidence on the association between electronic cigarette use and periodontal health, while also identifying important knowledge gaps and priorities for future research.

Although peri-implantitis is triggered by a polymicrobial biofilm, its progression is influenced by factors beyond microbial buildup. Another article in this Special Issue examines these factors and their implications for risk assessment, prevention, individualized management, clinical decision-making, and long-term treatment success.^8^ It synthesized peri-implantitis progression and its modulating factors, discussing their implications for risk assessment, prevention, individualized management, clinical decision-making, and long-term treatment success.

An additional growing area that warranted closer examination was digital dentistry. It has rapidly changed approaches to the rehabilitation of edentulous patients. One of the articles maps the available evidence on digital workflows for full-arch rehabilitation, identifying research trends, knowledge gaps, and implications for clinical-decision making. A structured synthesis of the current scenario of digital workflows for oral rehabilitation of edentulous patients is presented.^9^

Finally, this collection of articles invites researchers and clinicians to revisit established concepts and practices considering the best available evidence. More than simply bringing together reviews on contemporary subjects, this Special Issue seeks to stimulate critical reflection on widely held beliefs, promote *Dentistry Beyond the Myth,* and strengthen Evidence-based clinical research and practice.

Evidence alone, however, is never sufficient to determine the best course of action. Clinical decisions should integrate the best available evidence relating to the patient’s oral and medical condition and history with clinical expertise and the patient’s treatment needs, preferences, and circumstances through a shared decision-making process.^10^, ^11^

We hope that this initiative will also inspire new research questions and contribute to advancing dental science in the years to come.
