# Erratum: Evidence-based approaches for advancing clinical research and practice: dentistry beyond the myth

**DOI:** 10.1590/1807-3107bor-2026.vol40suppl1057err

**Published:** 2026-09-28

**In Editorial**

Lucianne Cople MAIA. Evidence-based approaches for advancing clinical research and practice: dentistry beyond the myth. Braz Oral Res. 2026;40(suppl 1):e057. https://doi.org/10.1590/1807-3107bor-2026.vol40suppl1057

**Where it reads:**

**In references 1 through 9, where it reads DOIS**

1. Visconti MA, Bornstein MM, Santos-Silva AR, Martins MD, Oliveira ML. Artificial intelligence for the detection and diagnosis of oral and maxillofacial pathologies: evidence, limitations, and future directions. Braz Oral Res. 2026;40 (suppl1):e058. https://doi.org/10.1590/1807-3107bor-2026.vol40.00058

2. Koth VS, Gabriel AF, Deckmann I, Castilho RM, Nor F Lamers ML, et al. Photobiomodulation in the management of oral lesions: a targeted, evidencebased approach. Braz Oral Res. 2026;40 (suppl1):e059. https://doi.org/10.1590/1807-3107bor-2026.vol40.0059

3. Soares IPM, Pires MLBA, Oliveira JR, Fernandes LO, Costa CAS, Bottino MC, et al. More than a trend: the rationale for using bioactive materials in clinical practice. Braz Oral Res. 2026;40 (suppl1):e60. https://doi.org/10.1590/1807-3107bor-2026.vol40.0060

4. Neves AA, Santos-Pinto L, Rios D, Soviero VM. Key concepts for clinical management of molar incisor hypomineralization. Braz Oral Res. 2026;40 (suppl1):e61. https://doi.org/10.1590/1807-3107bor-2026.vol40.0061

5. Reis A, Maran BM, Cordeiro DCF, Barbosa LMM, Terra RMO, Loguercio AD, et al.. Clinical myths in dental bleaching: an evidence-based structured narrative review. Braz Oral Res. 2026;40 (suppl1):e062. https://doi.org/10.1590/1807-3107bor-2026.vol40.0062

6. Risso PA, Portes-Zeno AP, Santos AG, Sousa ACS, Arruda-Vasconcelos R, Silva EJNL, Sipert CR. Regenerative endodontics: current concepts, clinical challenges, and emerging therapies. Braz Oral Res. 2026;40 (suppl1):e063. https://doi.org/10.1590/1807-3107bor-2026.vol40.0063

7. Pannuti CM, Silva TE, Aquino SG, Steffens JP, Nascimento GG, Leite FRM. E-cigarettes and periodontal health: current evidence and research gaps. Braz Oral Res. 2026;40 (suppl1):e64. https://doi.org/10.1590/1807-3107bor-2026.vol40.0064

8. Andrade CSAF, Silva JPS, Oliveira Júnior GO, Nagay BE, Shibli JA. Souza JGS, et al. Peri-implantitis progression and modulating factors: evidence synthesis and implications for clinical management. Braz Oral Res. 2026;40 (suppl1):e65. https://doi.org/10.1590/1807-3107bor-2026.vol40.0065

9. Carreiro AFP, Ribeiro AKC, Çakmak G, Lam WYH, Lin W-A, Park J-M, et al. Digital era in oral rehabilitation: a scoping review of trends and implications for clinical practice. Braz Oral Res. 2026;40 (suppl1):e66. https://doi.org/10.1590/1807-3107bor-2026.vol40.0066

**It should read:**

1.Visconti MA, Bornstein MM, Santos-Silva AR, Martins MD, Oliveira ML. Artificial intelligence for the detection and diagnosis of oral and maxillofacial pathologies: evidence, limitations, and future directions. Braz Oral Res. 2026;40 (suppl1):e058. https://doi.org/10.1590/1807-3107bor-2026.vol40suppl1058

2. Koth VS, Gabriel AF, Deckmann I, Castilho RM, Nor F Lamers ML, et al. Photobiomodulation in the management of oral lesions: a targeted, evidencebased approach. Braz Oral Res. 2026;40 (suppl1):e059. https://doi.org/10.1590/1807-3107bor-2026.vol40suppl1059

3. Soares IPM, Pires MLBA, Oliveira JR, Fernandes LO, Costa CAS, Bottino MC, et al. More than a trend: the rationale for using bioactive materials in clinical practice. Braz Oral Res. 2026;40 (suppl1):e60. https://doi.org/10.1590/1807-3107bor-2026.vol40suppl1060

4. Neves AA, Santos-Pinto L, Rios D, Soviero VM. Key concepts for clinical management of molar incisor hypomineralization. Braz Oral Res. 2026;40 (suppl1):e61. https://doi.org/10.1590/1807-3107bor-2026.vol40suppl1061

5. Reis A, Maran BM, Cordeiro DCF, Barbosa LMM, Terra RMO, Loguercio AD, et al. Clinical myths in dental bleaching: an evidence-based structured narrative review. Braz Oral Res. 2026;40 (suppl1):e062. https://doi.org/10.1590/1807-3107bor-2026.vol40suppl1062

6. Risso PA, Portes-Zeno AP, Santos AG, Sousa ACS, Arruda-Vasconcelos R, Silva EJNL, Sipert CR. Regenerative endodontics: current concepts, clinical challenges, and emerging therapies. Braz Oral Res. 2026;40 (suppl1):e063. https://doi.org/10.1590/1807-3107bor-2026.vol40suppl1063

7. Pannuti CM, Silva TE, Aquino SG, Steffens JP, Nascimento GG, Leite FRM. E-cigarettes and periodontal health: current evidence and research gaps. Braz Oral Res. 2026;40 (suppl1):e64. https://doi.org/10.1590/1807-3107bor-2026.vol40suppl1064

8. Andrade CSAF, Silva JPS, Oliveira Júnior GO, Nagay BE, Shibli JA. Souza JGS, et al. Peri-implantitis progression and modulating factors: evidence synthesis and implications for clinical management. Braz Oral Res. 2026;40 (suppl1):e65. https://doi.org/10.1590/1807-3107bor-2026.vol40suppl1065

9. Carreiro AFP, Ribeiro AKC, Çakmak G, Lam WYH, Lin W-A, Park J-M, et al. Digital era in oral rehabilitation: a scoping review of trends and implications for clinical practice. Braz Oral Res. 2026;40 (suppl1):e66. https://doi.org/10.1590/1807-3107bor-2026.vol40suppl1066

